# Design of planar and wideangle resonant color absorbers for applications in the visible spectrum

**DOI:** 10.1038/s41598-019-43539-2

**Published:** 2019-05-07

**Authors:** Igor Leonardo Gomes de Souza, Vitaly Felix Rodriguez-Esquerre

**Affiliations:** Department of Electrical Engineering, Federal University of Bahia, Salvador, 40155-250 Brazil

**Keywords:** Nanophotonics and plasmonics, Integrated optics

## Abstract

We propose a design approach for color absorbers based on a tri-layer metal-dielectric-metal (MDM) planar geometry, which maintains the same color absorbed, over a range of incident angles from 0° to 80° for light with TM polarization. The dielectrics are chosen to satisfy the ideal conditions of resonance. We calculate the ideal thickness of each dielectric layer by using the planar resonance theory. The numerical results show a total absorption above 85% for all colors of the absorber. We analyzed the influence of the of the metallic top layer thickness and we demonstrated the fabrication error tolerance of the proposed absorber. Finally, we present and discuss the physical mechanisms for the coupling of the electromagnetic field and the absorbed optical power in the structure.

## Introduction

Ideal absorbers are structures where there is no reflection or transmission of the incident electromagnetic field at any wavelength of interest^1^. Electromagnetic absorbers can convert the incident electromagnetic energy on their surface into heat or other forms of energy. They have attracted the researchers attention due to the possibility of being used in a wide range of applications such as thermal emission^2,3^, photovoltaic cells^4^, light coupling^5^, sensors^6^, photodetectors^7^ and solar thermoelectric generators^8^. Nowadays, many designs of absorbers have been proposed, fabricated and tested. Generally, these absorbers consist of metal/dielectric multilayers, they have a high loss, and they are bulky structures. They also exhibit low wavelength bandwidth flexibility limiting their applications^9^. In the last decades, the advancement of the knowledge of metamaterials (MMs) associated to plasmonic nanostructures has provided the possibility of controlling and manipulating the interactions of the electromagnetic field with matter, allowing the development of novel structures where the absorption, transmission and reflection of the incident field can be controlled^10^. The unique optical properties of photonic MMs are strongly dependent on the ideal choice of size, geometry, periodicity, and materials (metals and dielectrics) of the nanostructure^11^. It has been demonstrated that absorbers with close to unity absorption, with approximately zero transmission and reflection, can be obtained by using nanophotonic MMs with impedance close to that of the free space by considering dielectric/metal layers in their design over a metallic substrate^3,8,11^. Broadband absorbers based on MMs and planar nanoresonators with absorption higher than 80% for visible and infrared radiation have been analyzed and several designs have been proposed. They are metal-dielectric-metal (MDM) structures^12–17^ with only one or more types of metals^18,19^, they are composed by multilayer pairs of metal/dielectric^20^. The possibility of being able to control the properties of reflection, transmission and absorption in photonic MMs is of great interest for the development and design of perfect absorbers and resonant optical filters of transmission for the visible and infrared spectrum^21^. Perfect absorbers based on nanophotonic MMs that operate on the wavelength of the ultraviolet, visible and infrared electromagnetic spectrum, operate in the sub-wavelength regime and require, in most cases, great precision in the fabrication process due to the nanometric size of the structure dimension. One alternative to overcome the complex fabrication process of nanophotonic MMs would be the deposition of continuous thin films of metal/dielectric layers. This way of design does not require expensive neither complex fabrication techniques. The flat metal/dielectric/metal (MDM) multilayer structure is known as a Fabry-Perot (FP) type resonator and with the appropriate choice of metals, dielectric and layer thickness, it produces resonances that can be applied in several electronic optical devices, such as light transmitters^22–24^, photodetectors^25^, modulators^26^, phototransistors^27^ and photovoltaic cells^28^. FP filters face the challenge of shifting to blue in a similar way than other thin film based filters^23^ for oblique incident waves. The angular tolerance of FP resonators can be improved when specific conditions are previously satisfied^29^, especially the condition that relates the physical permittivity constants of the metal and the dielectric used in the design of the resonator. In this work we propose a planar FP absorber for all the colors of the visible spectrum. We use the theory of planar FP resonators to find the proper dielectric and its thickness for each specific color. We also evaluate the behavior of the absorbers for large angles of incidence and for possible fabrication errors of the metal thickness.

In summary, in this work we present the proposal of absorbers exploring the properties of a Fabry Perot resonator where the combined effects of the dispersion properties of the dielectrics and metals used in their design result in a wideangle narrowband resonator for incident angles of about 80°. The absorbers are designed for the whole spectrum of visible light. Six different dielectrics have been considered in our study to achieve the resonance conditions (*ε*_*dielectric*_ ≈ *εmetal*). In addition, we also evaluated the effects of fabrication errors in the deposition of the top layer of Ag and the physical mechanisms of absorption through the analysis of the spatial distribution of the electromagnetic fields inside the resonant cavity.

## Results and Discussions

**Figure 1:**
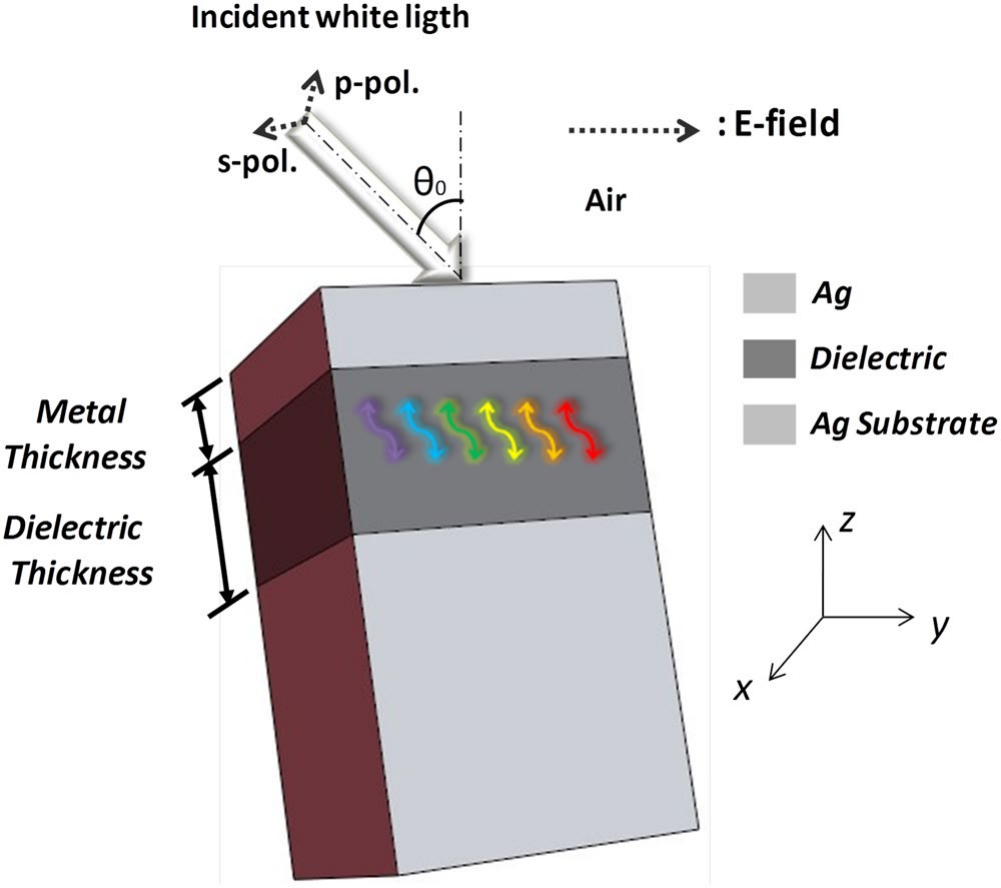
Proposed FP color absorber based on a nanoresonator integrated with a dielectric overlay.

**Figure 2:**
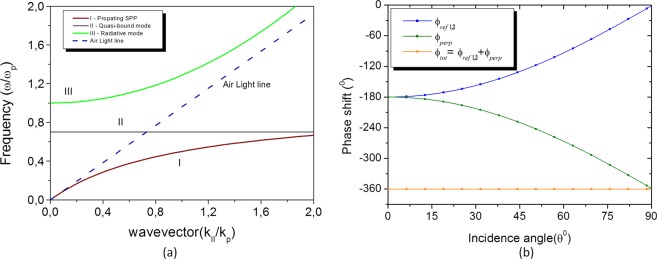
(**a**) The Dispersion curve of a MDM structure comprising two Ag mirrors separated by an air with the thickness of 0.25*λ*_*SP*_. The modes that are confined in the dielectric region are: I - Propagating SPP; II - Near-bound mode and III - Radiated mode; (**b**) the round-trip phase change in the planar resonator. The blue line shows the displacement of the reflection phase at the two interfaces and the green line shows the displacement of the propagation phase on the normal surface in the dielectric. The total displacement of the round-trip phase is represented by the orange line which is approximately 2π for all *θ*’s.

Figure 1 shows the proposed FP nanoresonator. It is composed by a planar tri-layer structure, consisting of a thin metal film at the top over a dielectric layer and a metal substrate. The chosen metal in the design was Silver (Ag) due to the low value of the real part of the permittivity in the visible range of the electromagnetic spectrum. The metal remains the same in all the filters in order to study the effect of dielectrics. The Fig. 2a shows the dispersion curve of the planar FP resonator, composed of two semi-infinite metal mirrors separated by 0.25*λ*_*sp*_. We use the frequency dependent permittivity of silver given by^30^. In addition, the metal and the dielectrics are non-magnetic *μ*_1_ = *μ*_2_= 1.

The modes that are confined in the dielectric region are: I - Propagating SPP; II - Near-bound mode and III - Radiated mode. The mode of interest to be analyzed is the Near-bound mode, because a significant part of its dispersion ratio is above the air line. Consequently, the external incident light can be coupled to the dielectric region depending on the thickness of the metal^21^. The dispersion relation of the planar resonator is defined as

1 $${k}_{//}=\omega {c}^{-1}{({\varepsilon }_{m}{\varepsilon }_{d}/\varepsilon {}_{m}+{\varepsilon }_{d})}^{1/2}$$

When *k*_//_ approaches infinity, *ω*(*k*_//_), the dispersion frequency as a function of *k*, tends to *ω*_*sp*_ which is the frequency of the surface plasmon. At this frequency the dielectric permittivity of the metal (*ε*_*m*_) and the permittivity of the dielectric *ε*_*d*_ are equal in magnitude but with opposite sign,

2 $${\varepsilon }_{dielectric}=-{\varepsilon }_{metal}\,{\rm{or}}\,|{\varepsilon }_{dielectric}|=|{\varepsilon }_{metal}|$$

This situation occurs when the thickness of the dielectric assumes the value of equation

3 $${d}_{dielectric}=\frac{{\lambda }_{SP}}{4\sqrt{{\varepsilon }_{dielectric}}}$$

*λ*_*sp*_ = 2*πc*/*ω*_*sp*_ is the wavelength of the free space surface plasmon, because the frequency in *k*_//_ = 0 will coincide with *ω*_*sp*_ then the dispersion band II can become almost completely flat. Furthermore, this plane dispersion range of mode II (Fig. 2a) indicates that the resonance at a given wavelength should occur approximately for all incident angles^23,24,31^. When Eqs (2) and (3) are satisfied due to the choice of the ideal metal and dielectric, light that strikes the planar nanoresonator will have a reflection phase shifting at the upper and bottom metal/dielectric interfaces, (*φ*_*ref*1_) and (*φ*_*ref*2_), respectively. This displacement cancels approximately with the phase shifting of the light propagating in the perpendicular direction (*φ*_*perp*_) to the interfaces in the dielectric region. The sum of the total shifting displacement in each round trip for all angles of incidence *θ* is approximately −2π^31,32^. This behavior can be observed in Fig. 2b.

**Figure 3:**
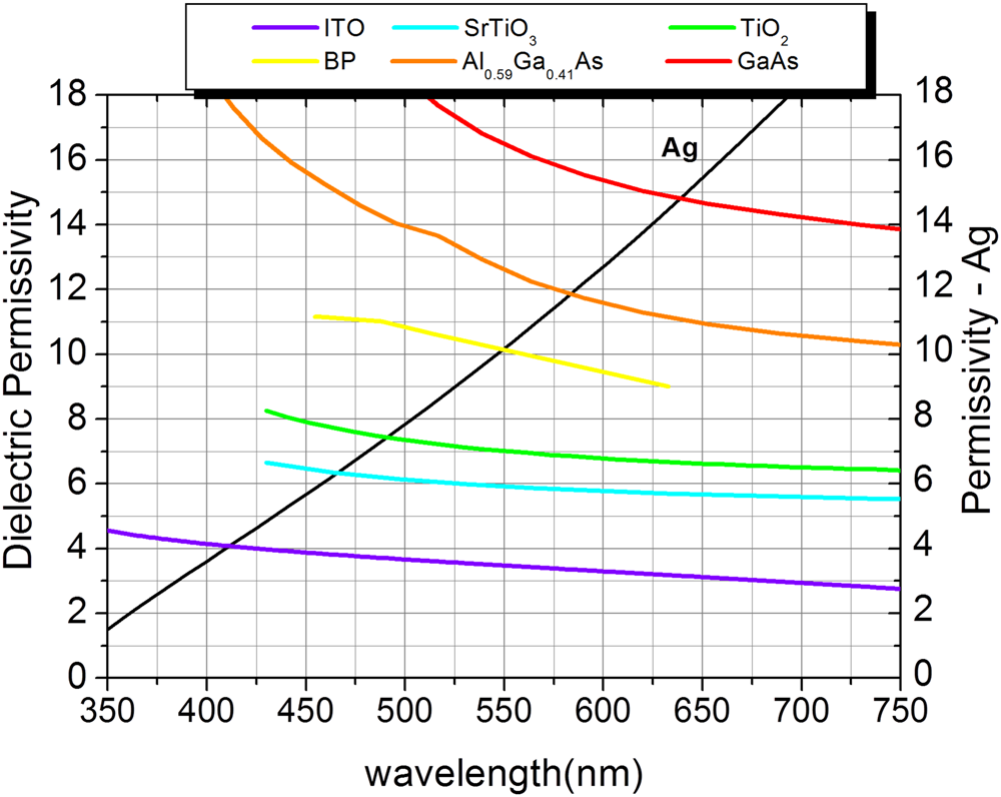
Relative Permittivity of Ag and of each dielectric used in the absorber resonant project.

The chosen metal for the nanoabsorber is Ag, the criterion for this choice is that in the visible region the real part of the permittivity is quite small and can be considered null^30,33^. Consequently, the relative permittivity can be described as ${\varepsilon }_{Ag}\approx i{\varepsilon }_{Ag}$, where $i{\varepsilon }_{Ag}$ is the imaginary part of the permittivity of Ag. Almost all dielectrics exhibit the imaginary part of the permittivity equal or very close to zero, consequently, only the real part of the dielectric permittivity has been considered, ${\varepsilon }_{dielectric}\approx {\varepsilon }_{dielectric,real}$. The theory for designing a planar structure that has angular insensitivity properties for the light with TM polarization predicts that Eq. 2 is satisfied^31^. Figure 3 shows the imaginary part of the relative permittivity of Ag in the region of the visible limited in the range of 350–750 nm. In this region Ag exhibits anomalous dispersion properties^23^. The imaginary part of the relative permittivity of Ag increases almost linearly in this interval with values varying from 1.6 to 18.0. Also, the relative permittivity of the selected dielectrics for the absorber design can be observed in Fig. 3. They satisfy the condition of FP absorbers insensitive to the angle $|{\varepsilon }_{dielectric}|=|{\varepsilon }_{metal}|$. The points of intersection of the permittivity curve of Ag and the curve of each dielectric satisfies the condition given by Eq. 2 for a perfect resonance in the interval of each color of the visible spectrum. Table 1 shows the theoretical parameters: color, wavelengths, bandwidth, ${\lambda }_{sp}$ which is the resonant wavelength given by the intersection of the permittivity curves of Ag and the dielectrics (Fig. 3) and the permittivity values at each intersection. These are the theoretical data of the design of the FP color absorber.

**Table 1:** Theoretical parameters of the angle insensitive absorbers in our study in accordance with Fig. 3.

Color	Bandwidth (nm)	Δλ_color_(nm)	λ_SP_ (nm)	Dielectric [ref]	ε_dielectric_ = ε_Ag_
Violet	380–450	70	411.0	ITO^38^	4.08
Blue	450–490	40	466.0	SrTiO_3_^39^	6.33
Green	490–540	50	491.0	TiO_2_^40^	7.44
Yellow	540–580	40	550.0	BP^41^	10.12
Orange	580–620	40	585.0	Al_0.59_Ga_0.41_As^42^	11.91
Red	620–750	130	639.0	GaAs^42^	14.81

**Figure 4:**
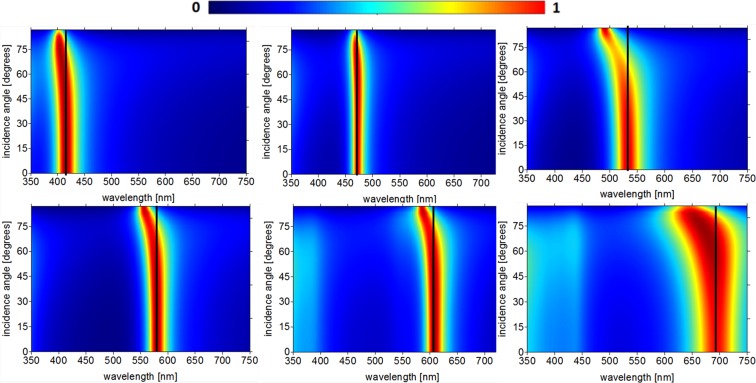
Dependence of the absorption for incident angles from 0° to 90° in the 350 to 750 nm range (**a**) violet(ITO), (**b**) blue(SrTiO_3_), (**c**) green(TiO_2_), (**d**) yellow(BP), (**e**) orange(Al_0.59_Ga_0.41_As) and (**f**) red(GaAs).

In order to calculate the absorption coefficients of the angle insensitive FP structure we used the Finite Element Method - FEM^34,35^, the results of the absorption dependence for the TM polarization can be observed in Fig. 4(a–f). The numerical results show near one unit absorption for all proposed absorbers for normal incidence (θ = 0^0^). It is also possible to observe that this high absorption is maintained in a wide interval of incident angles, from 0° to 60°, confirming the angle insensitive theory of FP absorbers. Figure 4a shows a small shifting of the absorption peak to the ultraviolet at angles greater than 50° but the resonance continues within the range of the color defined in Table 1. The blue absorber, Fig. 4b, exhibits the best result of the six proposed absorbers related to the wideangle operation. The resonant wavelength is almost the same as presented in Fig. 3 for all the incident angles and it corresponds to the central wavelength of the blue color. For the green absorber, Fig. 4c, a deviation for blue occurs at incident angles above 55°, the absorption is still within the color range 490–540 nm. From the results for the yellow and orange colors Fig. 4d,e it can be observed a blue-shifting for angles above 60°. Figure 4f shows the absorber for red color, also a blue-shift occurs at angles greater than 45°, but all the absorption spectrum is within the range of the red color defined in Table 1.

Table 2 shows the metal and dielectric thicknesses, the peak of the resonant wavelengths, the absorption and in the last column the full width at half-maximum (FWHM).

**Table 2:** Technical data and absorption results.

Color	d_dielectric_ (nm)	d_Ag_ (nm)	Peak – Abs [%]	FWHM(nm)
Violet	50.87	33	415–98%	35
Blue	46.32	46	472–91%	21
Green	45.00	24	533–92%	47
Yellow	43.23	31	580–99%	28
Orange	42.39	34	608–99%	32
Red	41.52	30	695–86%	82

**Figure 5:**
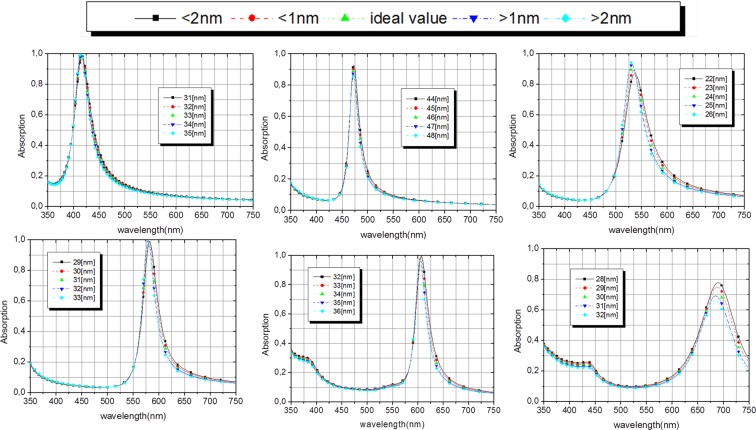
Dependence of the absorption with Ag thickness in the 350 to 750 nm range (**a**) violet, (**b**) blue, (**c**) green, (**d**) yellow, (**e**) orange and (**f**) red.

We use the intersection points *λ*_*sp*_ of each color shown in the Fig. 3 (and in the Table 1) and Eq. 3 to calculate the ideal thicknesses of each dielectric. The ideal values are in the second column of Table 2. We can note that all absorption peaks are within the range of each color and the absorption is above 90% for all colors except for the red. The FWHM are rather narrow and smaller than Δ*λ*_*color*_ showing that the absorber does not shift to the neighboring color spectrum. Moreover, the Ag thickness has a great impact on the optical properties of the proposed absorbers, influencing the resonant wavelengths and angular insensitivity characteristics^33^. When the top layer of Ag are sufficiently thick (above 30 nm), the angular insensitivity characteristics for the TM polarization will be obtained, however, it increases the reflection by decreasing the absorption at the resonant peak. On the other hand, if the thickness of the Ag layer decreases, the absorption increases for normal incidence, despite the absorption peaks are shifted to the neighboring spectral range for oblique incidence, losing the wideangle absorption characteristics. Several simulations have been carried out in order to find the ideal Ag thickness of the top layer and the designed absorbers exhibit maximum absorption and angular insensitivity. The optimized values are shown in the second column of Table 2. Although planar geometry is easy to fabricate and the processes can be controlled to have little or no error, we analyze the behavior of each narrowband absorber for a possible process of fabrication error. The Ag layer thickness has been increased and decreased in 2 nm from its ideal value. Figure 5 shows the tolerance analysis results for the six absorbers, it is possible to observe a good error tolerance and all the absorbers practically maintained their initial behavior.

**Figure 6:**
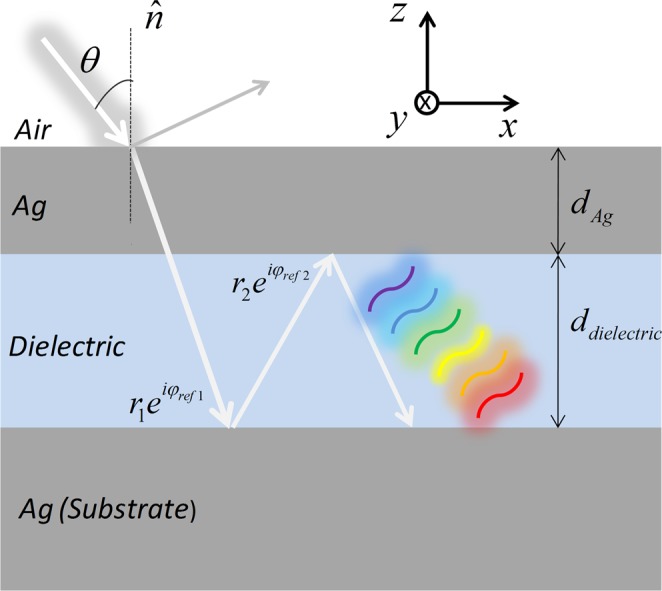
Physical coupling mechanism of the nano absorber FP, incorporating a phase compensation overlap, ${\phi }_{1}$ and ${\phi }_{2}$ are the reflection phase shift at the two metal–spacer interfaces, respectively.

Figure 6 shows the physical coupling mechanism of the nanoabsorber FP, according to^21^ when Eqs (2) and (3) are satisfied, the aggregate phase shift at the metal/dielectric interfaces (${\phi }_{ref1}+{\phi }_{ref2}$) is canceled with the phase shift in the perpendicular direction to the interfaces in the dielectric region ${\phi }_{perp}=4\pi {{\varepsilon }^{0.5}}_{dielectric}{d}_{dielectric}\,\cos \,\theta /\lambda$. The transmission is null when a thick metallic substrate is used and the perfect condition of constructive interference between the incident wave and the reflected wave is given by^36^

4 $${\phi }_{ref1}+{\phi }_{ref2}+4\pi {{\varepsilon }^{0.5}}_{dielectric}{d}_{dielectric}\,\cos \,\theta /\lambda =2m\pi $$

With $m=0,\pm 1,\pm \mathrm{2..}.$, when the conditions are satisfied according to the discussion here presented and when $\lambda \approx {\lambda }_{sp}$, total absorption of the incident light can be obtained.

**Figure 7:**
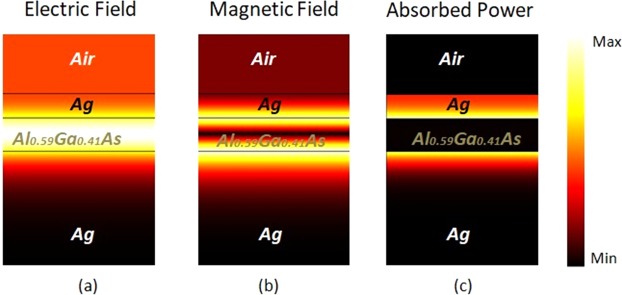
Spatial distribution of the (**a**) normalized Electric field, (**b**) normalized Magnetic field and (**c**) Absorbed power for the MDM absorber at the maximum absorption peak wavelength of 580 nm.

In order to understand and visualize the coupling mechanism of the FP absorber, we calculated the normalized electric field (*E*), normalized magnetic field (*H*) and the absorbed optical power (*P*_*abs*_) at the absorption peak of the yellow absorber, λ = 580 nm, Fig. 7(a–c). The *P*_*abs*_ was calculated by multiplying the electric intensity and the imaginary part of the permissiveness of the metal^37^:

5 $${P}_{abs}=\alpha .\text{Im}(\varepsilon ).{|E|}^{2}$$

where *α* is the normalized coefficient and the electric field *E* was calculated by FEM. Figure 7a shows the strong concentration of the *E-*field in the dielectric region between the metal film and the substrate, as a result, a standing wave is formed in that region. This occurs due to the constructive interference caused by the choice of the parameters to satisfy Eq. 4 for the phase compensation, resulting in total absorption at the resonant peak *λ*_*sp*_. The spatial distribution of the normalized magnetic field *H* is shown in Fig. 7b, it is possible to observe a strong intensity at the metal/dielectric interface. This can be attributed to the excitation of Surface Plasmons Polaritons (SPPs) that occur for the TM polarization. The strong coupling of *E* in the cavity makes *P*_*abs*_ to be absorbed inside the metallic film, both at the upper and lower interface (substrate), as can be seen in Fig. 2c.

## Conclusion

In conclusion, a narrowband resonant planar structure based on MDM films is proposed to achieve maximum absorption in the visible spectrum. The proposed absorber is insensitive to the angle of incidence for TM polarization. We used Ag as the metal in the design of the project because the real part of the relative permittivity is approximately zero in the visible spectrum. In addition, we chose six different dielectrics to satisfy the the Fabry-Perot resonance conditions. The numerical results show that the absorption is above 85% for all proposed absorbers. The dependence with the angle of incidence was also analyzed and it was possible to observe that the high absorption was maintained even in cases where a small blue-shift of the peaks is present and the peak wavelength still stays within the range of the color. It was possible to calculate the ideal dielectric thickness of each absorber by using the discussed theory. The excellent results did not change by varying the thickness of the metal to consider possible errors in the fabrication process. The physical absorbing mechanism is attributed to the reflection phase compensation, caused by the choice of the physical parameters (optical constants) and geometric (thicknesses in the metal and dielectric) of the structure. The proposed structure has a simple geometry and it is of easy fabrication. It only requires planar film deposition. The absorbers can be used in other regions of the electromagnetic spectrum as long as the discussed resonance conditions are satisfied. The proposed device can be used in optoelectronic devices including sensors, photodetectors, perfect absorbers, among others.
